# The Chicago Medical Society

**Published:** 1876-06

**Authors:** D. C. Stillians


### Reports of Societies.

### THE CHICAGO MEDICAL SOCIETY.

*Regular Meeting, Dec.* 6, 1875.

(Reported by D. C. STILLIANS, M.D.)

The President, Dr. Wm. E. Clark, occupied the chair.

Dr. T. D. Fitch reported a case of retro-versio uteri cured by restoration, mechanical support and pregnancy.

Mrs. R., aged twenty-two years, married five years, mother of one child aged two years (living), since the birth of which she has never conceived—never had an abortion.

Patient began to menstruate at the age of sixteen. Always enjoyed robust health up to the time of her only confinement, when she was attended by a midwife, and rose from her bed in four days. She states that she has "never seen a well day since." From personal observation I can verify the fact of her previous good health.

During the time which has elapsed since her confinement, she has suffered from the following symptoms: Constant pain in the lumbo-sacral region, with a sense of bearing down and weight in the pelvic organs; constant and obstinate constipation, with occasional attacks of rectal tenesmus ; pain extending down the thighs, rendering locomotion difficult ; almost constant vesical tenesmus ; a frequent desire to micturate, attended with more or less pain ; dyspepsia, with acid eructations, anorexia, nausea, and sometimes vomiting, with consequent progressive emaciation.

Menstruation has been somewhat irregular, both as to time and character—sometimes occurring every twenty days, sometimes twenty-eight days, the former time the rule, however. She has been menorrhagic, the discharge being excessive and partially coagulated. The epochs were usually attended with evidences of pathological congestion, such as pain and fever. Leucorrhœa of a muco-purulent character has been constantly present, though not accompanied by profuseness of discharge.

On the 17th of March, 1871, the physical signs were as follows :

In the dorsal decubitus, (which I prefer) the touch revealed a large ostium vaginæ, the urethra thickened and greatly hypertrophied, and a round, smooth and hard body in the axis and at the distal extremity of the vagina. Not finding the cervix, and presuming this body to be the uterus, my finger was passed along the anterior wall of the vagina (posterior wall of the uterus), and the cervix was felt immediately behind the pubic arch, and impinging strongly on the basfond of the bladder and the urethra.

The uterus was sensitive to pressure, and movable, indicating the absence of adhesions.

On conjoined manipulation, the body of the uterus was found in abnormal position. The speculum revealed eversion of the labia uteri, with granular erosion and patulous os.

To the sound there was hyperæsthesia of the cervical canal. It passed in a downward and backward direction to the extent of 3½ inches, some pain being elicited by pressure upon the fundus. No blood followed its removal, and the pain being transient, no metritis or endometritis was supposed to exist.

*Diagnosis.*—Retro-version, with chronic endo-cervicitis.

*Treatment.*—The indications were to restore the uterus to its normal position, and retain it there, at the same time to administer such remedies as would relieve the gastric trouble, and to employ such applications as would subdue the endocervicitis and heal the erosions. Believing in the primary restoration of the uterus, and that then, without material treatment, the evil results of a malposition will cease, I take this to be the first step in the course of the treatment. If we should adopt the plan of removing the first factor in the series of causes and effects, the subinvolution should be first treated. But it would be impossible to cure this hypertrophy while the circulation is so embarrassed by the distortion of the blood-vessels supplying the parts. In the majority of cases, on restoring the organ and thereby relieving the embarrassed circulation, the congestion and enlargement, together with the other evil results, disappear spontaneously. I therefore touched the granular surface of the cervix and the cervical canal with a saturated solution of iodine in pure creosote, and introduced a cotton tampon saturated with a solution of tannic acid in pure glycerine, in the proportion of ʒ iij of the acid to ℥ j of glycerine, for the purpose of relieving irritation, and hardening and accustoming the parts to the presence of a foreign body.

A pill containing two grains of the sulphate of quinine with one of the sulphate of iron, was also given before each meal, and a powder containing eight grains of the subnitrate of bismuth, and four of pepsine, was ordered after each meal.

In four days the tampon was removed, and the parts thoroughly washed with warm water by means of the uterine Anssing syringe; another tampon was then introduced. The patient expressed herself as feeling somewhat improved, in two days more, the tampon giving her great comfort and support; when it was removed and the parts washed as before, there was no apparent soreness or tenderness. The dislocation was then reduced by manipulation with the patient in the lateral decubitus (Sims'). The index finger was introduced into the vagina, pressing it against the body of the uterus, as near the fundus as possible, carrying it upward and forward in the arc of its descent. When carried as high as possible, it was held in this position for a few minutes, so as to give time to change the pressure from this point to the anterior lip of the cervix, which was carried downward and backward in the concavity of the sacrum higher up, if possible, than its normal position. It was held for two or three minutes that it might become accustomed to its new place, thus preventing its return before the pessary could be introduced. The latter was a modification of Hodges' horse shoe pessary, selected on account of the hypertrophied condition of the urethra. Its arms were partially straightened on account of the fullness of the post vaginal region. No discomfort was experienced by the patient on arising, and in fact there was no consciousness of the presence of a foreign body in the vagina.

A solution was ordered for vaginal injection, to be used once or twice daily, in order to keep the parts clean and prevent accumulation of the secretions behind the instrument, thus endangering irritation, ulceration, etc.

Patient expressed herself as much improved in every particular, six days afterward. To the erosions and cervical canal the iodo-creosote mixture was again applied.

April 4. Improvement continues in every particular. No inconvenience from pessary after removal. The points upon which it had pressed were found entirely healthy. It was reintroduced after applying the creosote mixture.

The patient was treated in the same manner on the 14th,

21st, and 28th, and May 3rd, and 23rd. At this last date it was found that she had just passed her menstrual period without the flow. Her improvement was so marked that I correctly supposed her to be pregnant. The pessary was worn and the injections continued till the end of the 3rd month, when it was removed, but the patient returned next day, complaining of the loss of its support, and a sensation of weakness. The pessary was again introduced and allowed to remain till the middle of the 5th month, when it was finally removed. She was at last delivered of a healthy boy.

In order that involution might be well completed before she arose from bed, I kept her in the recumbent position for three weeks, when she arose without the recurrence of the displacement. She has now remained free from uterine disease for a period of four and a half years.

The subject was discussed by Drs. Quine, Foster, and others.

A partial Report of the Section on Practical Medicine —Diseases of the Chest—was presented by E. Fletcher Ingals, M.D., as follows:

Since the appointment of this Section there has been no great advance in that part of practical medicine relating to diseases of the chest. Our theories regarding their causes and pathology have experienced no marked alteration; our methods of diagnosis have not been noticeably improved, and the elements of prognosis remain unchanged.

In treatment there has been some advancement, not so much in the discovery of new methods, as in the more general application of old ones.

I have observed in one instance an anomalous sign, in connection with consolidation of the lung, consisting of numerous fine blowing or whistling sounds resembling sibilant rales; these were heard over the apex of the right lung, and were at first supposed to be fine bronchial rales, but directly their rhythm pointed to their source; for they occurred not only during inspiration and expiration but also in the period of rest following the respiratory act. They were, in fact, independent of the air in the bronchial tubes and were synchronous with the contraction of the heart, as was easily demonstrated by directing the patient to hold his breath. Though no other auscultatory signs were present at the time, I believed this to be the result of pressure, by tuberculous deposits, upon several small arteries within the lung. A few weeks after my examination, the patient died of some intercurrent affection and I had an opportunity of verifying my diagnosis by a post-mortem examination, which revealed tuberculous consolidation in the apex of the right lung.

Surgeons enjoin rest in the treatment of any acutely inflamed organ ; but until lately physicians have too much disregarded this rule in treating pulmonary affections. In pleuritis and pneumonia much benefit will be effected by keeping the patient quiet, diminishing as much as possible, consistently with good aeration of the blood, the frequency of respiration, and by limiting movements of the affected side. The same course of treatment will often be beneficial in pleurodynia, pneumothorax and emphysema; and sometimes in phthisis. In the acute diseases mentioned, whether the inflammation be trifling or considerable, the patient should be put to bed and kept quiet until he recovers. Coughing should generally be checked, all exertion prevented, talking prohibited ; indeed, the patient must do nothing to waste breath.

Respiratory movements may be restrained to a considerable extent by the patient's voluntary efforts, if his attention is only drawn to the matter. He should be furnished good air and in some cases, especially of pneumonia and bronchitis, it is best saturated with watery vapor.

The room should be kept well ventilated and at a moderately high temperature. Where moisture is desired, steam may be introduced into the apartment. Breathing compressed air has been found to lessen the frequency of respiration ; it is, therefore, recommended in some cases, though unfortunately it can not often be employed for want of conveniences. It may be that in pleuritis and pneumonia, benefit would be derived from the respiration of pure oxygen, because of the longer rest which would thereby be afforded the inflamed organ.

By external appliances we may restrict the movements of the affected side, with the effect of greatly relieving the patient's sufferings, and, doubtless, shortening the course of the disease.

In simple pleurisy and pneumonia we may accomplish this end by adhesive plaster applied as directed by Dr. F. T. Roberts, in the *London Practitioner.* His method is, to apply two or three layers as follows: "The first strip is laid on obliquely in the direction of the ribs ; the second across the course of the ribs; the third in the direction of the first, about half overlapping it; the fourth as the second ; and so on, until the entire side is covered. A strip is also passed over the shoulder, which is kept down by another fixed round the side across its ends." The transverse strips should reach from the spine to the mesian line in front, and all should be applied if possible during full expiration. It is sometimes desirable to fix the chest in inflexible dressings, as in pneumothorax and fractures of the ribs ; for this we may employ plaster of Paris or any similar dressing such as surgeons use in making immovable splints. In emphysema with bulging chest walls, I would expect benefit from a nicely fitting elastic jacket ; which would in some measure compensate for the loss of elasticity in the lungs. Unless too great pressure were made, this appliance could not harm the thoracic walls ; it would rather, by its resistance, induce hypertrophy of the inspiratory muscles, and by its elasticity aid the expiratory, in their difficult task of expelling vitiated air from the lungs.

It seems reasonable to hope that this appliance would ultimately reduce the emphysematous chest in size, and greatly facilitate the patient's breathing. At first the pressure must be slight, but as the muscles become stronger it may gradually be increased. The jacket should be made to lace both in front and behind, so that the pressure may be regulated at will.

Paracentesis-thoracis has been practiced since the days of Hippocrates, but its popularity has been greatly increased by the recent introduction of improved aspirators. This operation in olden times was the *dernier ressort;* but through the teachings of Trousseau, Bowditch, Gairdner and others it became the common practice to employ the trochar as soon as other remedies had failed to produce absorption. With recent instruments the operation has become so easy, both for physician and patient, that we are reluctant to await even the action of diuretics and purgatives, and are almost tempted to perform paracentesis without allowing nature an opportunity to remove the fluid. However, this like all other extremes should be avoided, and the operation confined to those cases which are not curable by simpler remedies. Where a fair trial of diuretics and purgatives has been unavailing, and in all cases in which the fluid occasions urgent dyspnœa, the operation should be unhesitatingly performed. The aspirator may be employed with comparative safety in removing fluid from the pericardial sac, but we must not forget that physicians of large experience have punctured a dilated heart, in consequence of a mistake in diagnosticating this condition.

Experiments have been made with the aspirator in treating pulmonary cavities. The cavities were punctured through the chest wall, their contents withdrawn, and remedies injected calculated to alter their surfaces so as to promote the healing process; but, as might have been expected, these experiments have proven unsatisfactory. Even if the cavities could thus be induced to heal, it is questionable whether the procedure would often be justifiable, in view of the danger from injury to the blood vessels, and of the possibility of establishing pneumothorax. When we remember that, in the majority of cases, pulmonary cavities result from a constitutional and not a local disease, we are surprised that good results could have been expected from this treatment.

I have recently seen a large aneurism of the abdominal aorta in which no murmur was present during the life of the patient. The tumor sprang from the anterior surface of the vessel between the diaphragm and cœliac axis, and protruded in the left hypochondriac region. It had a forcible heaving impulse with each cardiac systole. Death occurred about thirty-six hours after rupture of the sac.

